# Knowledge, attitude and practice towards physical activity among healthcare students at a public university in Saudi Arabia

**DOI:** 10.3389/fpubh.2024.1428165

**Published:** 2024-09-30

**Authors:** Geetha Kandasamy, Mona Almanasef, Tahani Almeleebia, Khalid Orayj, Lingala Kalyan Viswanath Reddy, Eman Shorog, Asma M. Alshahrani, Kousalya Prabahar, Vinoth Prabhu Veeramani, Palanisamy Amirthalingam, Saleh F. Alqifari, Rajalakshimi Vasudevan, Shaden Hamad AlSaedi, M. Yasmin Begum, Rehab Ahmed

**Affiliations:** ^1^Department of Clinical Pharmacy, College of Pharmacy, King Khalid University, Abha, Saudi Arabia; ^2^Department of Public Health, College of Health Sciences, Saudi Electronic University, Abha, Saudi Arabia; ^3^Department of Clinical Pharmacy, College of Pharmacy, Shaqra University, Dawadimi, Saudi Arabia; ^4^Department of Pharmacy Practice, Faculty of Pharmacy, University of Tabuk, Tabuk, Saudi Arabia; ^5^Department of Pharmacology, College of Pharmacy, King Khalid University, Abha, Saudi Arabia; ^6^College of Medicine, Taibah University, Madinah, Saudi Arabia; ^7^Department of Pharmaceutics, College of Pharmacy, King Khalid University, Abha, Saudi Arabia; ^8^Department of Natural Products and Alternative Medicine, Faculty of Pharmacy, University of Tabuk, Tabuk, Saudi Arabia

**Keywords:** physical activity, university students, healthcare, knowledge, attitude, practice

## Abstract

**Objective:**

This research aimed to study the knowledge, attitudes, and practices (KAP) toward physical activity (PA) of healthcare students at King Khalid University in Abha, Saudi Arabia.

**Methods:**

A prospective web-based cross-sectional study was carried out among healthcare students of King Khalid University from March to May 2024. The questionnaire contained questions on sociodemographic details, five questions about knowledge, eight questions about attitudes, and seven practice-related questions. A multivariable logistic regression analysis was used.

**Results:**

Out of the 383 participants, 175 (45.7%) were men and 208 (54.3%) were women. Most of the students, 292 (76.2%), had a body mass index (BMI) of 18.5–24.9. Among the 383, 264 (68.9%) showed good knowledge, 288 (75.2%) had positive attitudes, and 242 (63.2%) had satisfactory PA practice. Most of them, 310 (80.9%), were aware that “regular exercise helps promote strong bones and muscles.” Three hundred and fifty-two (91.9%) students believed that individuals who frequently engaged in PA had longer lifespans. More than half of the students (*n* = 239, 62.4%) engaged in the recommended level of PA. Of the students, 179 (46.8%) agreed that they planned to begin increasing their physical activity during the next 6 months. One hundred and fifty-six (40.7%) stated that they did sports with a high level of intensity for 20 min, three to five times a week, and 175 (45.7%) stated that they did moderate-intensity sports for 30 min, more than five times a week. None of the independent variables had a significant effect on good knowledge levels (*p* > 0.05). The regression results show being male OR = 0.473 (CI 0.285–0.783 *p* = 0.004), and being a first-or third-year student OR = 0.329 (CI 0.130–0.832 *p* = 0.013), OR = 0.276 (CI 0.100–0.762 *p* = 0.019) has a significant effect on positive attitude levels, while being male OR = 1.945 (CI 1.207–3.135 *p* = 0.006) and having a BMI between 18.5 and 24.9 OR = 10.101 (CI 2.104–48.49 *p* = 0.004) has a significant effect on satisfactory practice levels.

**Conclusion:**

The majority of participants in our study showed good knowledge and positive attitudes toward PA. A lower number of participants, though still the majority, had satisfactory levels of PA. Health education and health promotion initiatives should maintain and enhance knowledge of the positive effects of PA on health.

## 1 Introduction

Physical activity (PA) is a major factor in determining one’s level of physical fitness and health. PA is defined as any skeletal muscle-produced movement that requires energy expenditure (1). Noncommunicable diseases (NCDs) are medical conditions that are not caused by infectious agents and cannot be transmitted from one person to another. According to the World Health Organization (WHO), these diseases are characterized by their chronic nature and generally progress slowly over time. NCDs include cardiovascular diseases, cancers, chronic respiratory diseases and diabetes (2). PA is also known to prevent many of the world’s most common non-communicable diseases; healthcare students are taught about these diseases and the role PA plays in preventing them in their curriculum. Some healthcare students participate in various PAs every day, while others are unable or unwilling to do so for a variety of reasons, including lack of personal motivation, lack of access, health conditions, time management, cultural and social factors, and academic focus (3).

Recent studies have demonstrated the negative effects of physical inactivity on economies globally (4–6), which exacerbate the harm to the health and well-being of the general public. According to Guthold et al. (7), one of the biggest worldwide issues of 2016 was physical inactivity, putting over 1.4 billion people at risk of illnesses linked to it. Studies have continuously shown low levels of PA among the population, particularly in Saudi Arabia (8–11). According to the results of the most recent national survey carried out in 2019 by the Saudi General Authority for Statistics, 78% of Saudis were not engaging in PA (12).

Globally, inactivity is thought to be the cause of 9% of premature mortality, or over 5.3 million deaths each year (13). PA has a well-established history of lowering cardiovascular risks, improving lipid profiles, managing type 2 diabetes, preventing the occurrence of some malignancies, decreasing overall mortality, increasing psychological health and well-being, and increasing bone density (14). In 2013, the World Health Assembly decided to reduce insufficient PA globally by 10% voluntarily by 2025 (15). During their college years, people are more likely to face stress and time constraints, which may make it challenging for them to form healthy behaviors (16).

A few generations ago, Saudi Arabians led basic yet active lives. Back then, the physical demands of their hard jobs and everyday lives were enough to keep them at a suitable level of physical fitness and allow them to maintain a lean body mass (17). However, Saudi Arabia has experienced tremendous economic growth and affluence over the past few decades, along with a technological revolution that has significantly negatively impacted people’s lifestyles. Sedentary lifestyles, a diet high in calories, and the consumption of sugar-sweetened beverages has increased and spread throughout Saudi culture (18).

Studies show that people do not participate in PA frequently or for sufficient periods of time. This could be due to a variety of factors, including ignorance, and a lack of time, resources, and motivation (19). Furthermore, research on physical inactivity and its consequences in the Kingdom of Saudi Arabia is lacking (8). Hence, managing overweight and obesity may be aided by having the proper knowledge, attitude, and practice (KAP) regarding frequent PA. Nonetheless, KAP is greatly impacted by family support, caregiving responsibilities, learning readiness, socioeconomic and cultural factors, and learning environment (20). So, the goal of this study was to investigate the current KAP toward PA among healthcare students at King Khalid University in Abha, Saudi Arabia.

## 2 Methods

### 2.1 Study design and study site

A prospective cross-sectional study (web-based survey) was carried out among King Khalid University students from March to May 2024.

### 2.2 Population criteria

Inclusion criteria: King Khalid University healthcare students (dentistry, pharmacy, medicine, applied health sciences, and nursing) were enrolled in the study. Informed consent was written in the Google form. It is mentioned that participation in this study is voluntary at any time they can withdraw without penalty. Completing the questionnaire will confirm that the participant agrees to participate in the study. Therefore, each participant was clear about participating in the study.

Exclusion criteria: Students from outside the university, people who did not complete their questionnaires and people who did not give their consent to participate in the study were excluded from the research.

### 2.3 Sampling procedure and sample size

Using a snowball sampling technique, study participants were selected and enrolled. Snowball sampling was chosen to enhance the study’s ability to reach and recruit healthcare students at various department effectively, leveraging their existing networks and connections to achieve a representative sample for the study. The survey was made with Google Forms and shared online through social media and other channels. Participants completed an online survey. The Raosoft online sample size calculator was used to calculate the sample size with a 5% margin of error and a 95% confidence level of error. The overall number of healthcare students at KKU is approximately 1,250. To reduce the possibility of inaccurate results and improve the study’s reliability, a target sample size of *n* = 295 was determined. Overall, 421 participants initially responded, and 38 candidates were disqualified for incomplete information. The overall response rate of participants was 30.64%.

### 2.4 Questionnaire

The questionnaire was divided into four sections, the first of which asked questions about sociodemographic factors such as age, gender, year of study, and marital status. Body mass index (BMI), on the other hand, is used to assess an individual’s body weight relative to their height. There were five knowledge-related questions in Section 2, eight attitude-related questions in Section 3 (21, 22), and seven practice-related (23) questions in Section 4. The five knowledge questions had two possible scores: “correct” and “incorrect.” For knowledge, a correct response was valued at one mark, while an incorrect response was valued at zero. After combining them, the knowledge responses were evaluated. The knowledge score was further divided into low (≤3) or high (≥4) categories, as set out by Almutairi et al. (22).

A five-point Likert scale was applied to obtain responses about attitudes and practices. The participants were asked to rate their attitudes and practices on a scale of 1–5, with 5 denoting strong disagreement, 4 disagree, 3 neutral, 2 agree, and 1 strongly agree. The answers were gathered and assessed. The negative items were reverse coded (scoring range: 40–8, with 40 indicating the most positive attitude) and the total scores were calculated. Less than 60% were considered poor knowledge, negative attitude, and unsatisfactory practice, respectively. The Cronbach’s alpha test for the questionnaire’s general PA-related attitude questions was 0.73, and the questionnaire was validated in an earlier study (22).

### 2.5 Ethics approval

This research was approved by the King Khalid University Ethics Committee for the College of Pharmacy (HAPO-06-B-001; Approval No. ECM ≠ 2024-405).

### 2.6 Statistical analysis

IBM SPSS Statistics, a statistical tool for social sciences, version 21.0 for Windows, was used to conduct the statistical study. In the statistical analysis, both descriptive and inferential statistics were employed. The research participants’ sociodemographic data was characterized using frequency and percentages. To find out if there was a significant relationship between the independent and dependent variables, multivariable logistic regression analysis was done. Every test was two-tailed, with a significance level of *p* < 0.05.

## 3 Results

### 3.1 Descriptive results

Of the 383 participants, 208 (54.3%) were female and 175 (45.7%) were male. Out of the 383, 205 (53.5%) were between the ages of 18 and 20, 167 (43.6%) were between the ages of 21 and 25, and 11 (2.9%) were older than 25. In terms of marital status, the vast majority of the students, 356 (93%), were single, while 27 (7%) were married. Of the participants, 75 (19.6%) were first-year students, 73 (19.1%) were second years, 80 (20.9%) were third years, 83 (21.7%) were fourth years, and 72 (18.8%) were fifth years. In terms of body mass index (BMI), 22 (6.3%) were less than 18.5, 292 (76.2%) were between 18.5 and 24.9, 52 (13.6%) were between 25 and 29.9, and 15 (3.9%) were > 30. Details are in Table 1. The majority of participants, 264 (68.9%), showed good knowledge of PA, while 119 (31.1%) had poor knowledge. 288 (75.2%) had a positive attitude toward PA, while 95 (24.8%) had a negative attitude. 242 (63.2%) had satisfactory PA practice, while 141 (36.7%) had unsatisfactory PA practice. Details are provided in Table 2.

**Table 1:** Demographic details of study population.

Variables	Category	Number (%)
Gender	Male	175 (45.7)
	Female	208 (54.3)
Age	18–20 years	205 (53.5)
	21–25 years	167 (43.6)
	Above 25 years	11 (2.9)
Marital status	Single	356 (93)
	Married	27 (7)
Year of study	First year	75 (19.6)
	Second year	73 (19.1)
	Third year	80 (20.9)
	Fourth year	83 (21.7)
	Fifth year	72 (18.8)
BMI	<18.5	24 (6.3)
	18.5–24.9	292 (76.2)
	25–29.9	52 (13.6)
	>30	15 (3.9)

BMI, Body mass index.

**Table 2:** Scores of knowledge, attitude and practice toward physical activity among the healthcare students of university.

Knowledge	Number (%)
Good knowledge	264 (68.9)
Poor knowledge	119 (31.1)
**Attitude**	
Positive attitude	288 (75.2)
Negative attitude	95 (24.8)
**Practice**	
Satisfactory	242 (63.2)
Unsatisfactory	141 (36.8)

#### 3.1.1 Knowledge of PA

Of the 383 students, most of them, 310 (80.9%), were aware that “regular exercise helps promote strong bones and muscles.” 284 (74.2%) students concurred that obesity may be prevented with regular PA, and 269 (70.2%) were aware that engaging in regular physical activity lowers one’s risk of disease. 279 (72.8%) knew that exercise could help lower stress. According to 269 students (70.2%), sitting for longer than 2 h is harmful to muscles and bones. Details are provided in Table 3.

**Table 3:** Knowledge toward physical activity among healthcare students of university.

Questions (5)	Correct number (%)	Incorrect number (%)
1. Regular physical activity can help build strong bones and muscles	310 (80.9)	73 (19.1)
2. Regular physical activity can prevent obesity	284 (74.2)	99 (25.8)
3. Regular physical activity leads to a lower risk of diseases	269 (70.2)	114 (29.8)
4. Physical activity can reduce stress	279 (72.8)	104 (27.2)
5. Sitting for more than 2 h is bad for bones and muscles	269 (70.2)	114 (29.8)

#### 3.1.2 Attitude toward PA

Three hundred and fifty-two (91.9%) students believed that individuals who frequently engage in physical activity had longer lifespans than those who do not. Three hundred and thirty-nine (88.5%) students agreed that PA is something that everyone should do. One hundred and thirty-seven (35.7%) students disliked participating in PA, 195 (50.9%) liked to participate in it, and 51 (13.3%) were uncertain. Of the students, 136 (35.5%) agreed that girls should not engage in physical exercise, 213 (55.6%) disagreed, and 34 (8.9%) were unsure. Three hundred and eight (80.4%) agreed that regular participation in PA makes them healthy. 346 (90.3%) believed that PA can be used to manage the increased likelihood of certain diseases, such as diabetes, hypertension, and cardiovascular disease. Three hundred and thirty-eight students (88.2%) reported that engaging in physical activity made them feel happy. Regular PA improves blood circulation, according to 346 (75.2%) students. Details are provided in Table 4.

**Table 4:** Attitudes toward physical activity among healthcare students of university.

Questions	Strongly agree number (%)	Agree number (%)	Neutral number (%)	Disagree number (%)	Strongly disagree number (%)
1. People who regularly participate in physical activity live longer than those who do not	220 (57.4)	132 (34.5)	23 (6)	2 (0.5)	6 (1.6)
2. Everyone should participate in physical activity	233 (60.8)	106 (27.7)	31 (8.1)	8 (2.1)	5 (1.3)
3. I dislike participating in physical activity	61 (15.9)	76 (19.8)	51 (13.3)	49 (12.8)	146 (38.1)
4. Girls should not participate in physical activity	93 (24.3)	43 (11.2)	34 (8.9)	57 (14.9)	156 (40.7)
5. Regular participation in physical activity makes me healthy	208 (54.3)	100 (26.1)	41 (10.7)	16 (4.2)	18 (4.7)
6. Physical activity can be used to manage the higher risk of some diseases such as heart disease, diabetes, and high blood pressure	265 (69.2)	81 (21.1)	17 (4.4)	14 (3.7)	6 (1.6)
7. When I participate in physical activity, I feel happy	236 (61.6)	102 (26.6)	36 (9.4)	4 (1)	5 (1.3)
8. Regular physical activity increases blood circulation	263 (68.7)	83 (21.7)	25 (6.5)	6 (1.6)	6 (1.6)

#### 3.1.3 Practice toward PA

Over half of the students, 239 (62.4%), engaged in the recommended level of PA, with 79 (20.7%) not engaging in the recommended level, and 65 (17.0%) uncertain. Two hundred and forty students (62.4%) stated that they were unable to participate in physical exercise due to a medical condition. One hundred and ninety-nine students (52%) agreed that they exercised for enjoyment in addition to health benefits, 135 (35.2%) were unsure, and 49 (12.8%) disagreed. One hundred and seventy-nine students (46.8%) said they planned to begin increasing their physical activity during the next 6 months, compared to 133 (34.7%) who disagreed and 71 (18.5%) who were unsure.

One hundred and sixty-five students (43.1%) agreed with the statement “I get all the exercise I need from just doing normal activities,” 78 (20.4%) were unsure and 148 (38.6%) disagreed. One hundred and fifty-six students (40.7%) stated that they play sports with a high level of intensity (such as soccer, running, or aerobics) for 20 min, three to five times a week, 78 students (20.4%) were unsure, and 149 students (38.9%) disagreed. 175 (45.7%) agreed, 78 (20.4%) were unsure, and 130 (33.9%) disagreed that they do moderate-intensity sports (such as dancing, brisk walking, or light exercise) for 30 min, more than five times a week. Details are provided in Table 5.

**Table 5:** Practices toward physical activity among healthcare students of university.

Questions (5)	Strongly agree *n* (%)	Agree *n* (%)	Neutral *n* (%)	Disagree *n* (%)	Strongly disagree *n* (%)
1. I engage in the right amount of physical activity	91 (23.8)	148 (38.6)	65 (17.0)	60 (15.7)	19 (5)
2. I have a medical condition that prevents me from engaging in physical activity	108 (28.2)	132 (34.5)	100 (26.1)	35 (9.1)	8 (2.1)
3. I engage in exercise for pleasure, not only for health benefits	83 (21.7)	116 (30.3)	135 (35.2)	33 (8.6)	16 (4.2)
4. I intend to start doing more physical activity in the next 6 months	106 (27.7)	73 (19.1)	71 (18.5)	94 (24.5)	39 (10.2)
5. I get all the exercise I need from just doing normal activities	90 (23.5)	75 (19.6)	70 (18.3)	56 (14.6)	92 (24)
6. I engage in vigorous intensity sports for 20 min, 3–5 times a week (e.g., running, soccer, aerobics)	87 (22.7)	69 (18.0)	78 (20.4)	92 (24)	57 (14.9)
7. I engage in moderate-intensity sports for 30 min, more than 5 times a week (e.g., brisk walking, dancing, light exercise)	98 (25.6)	77 (20.1)	78 (20.4)	84 (21.9)	46 (12)

#### 3.1.4 Multiple logistic regression analysis

#### 3.1.5 Knowledge toward PA

When compared to the reference age group of over 25 s, the odds of having a good knowledge level decrease by approximately OR = 0.936 (CI 0.166–5.271 *p* = 0.940) and OR = 0.851 (CI 0.155–4.670 *p* = 0.853) for those between the ages of 18 and 20 and between 21 and 25, respectively. However, since *p* > 0.05, this difference is not statistically significant. Marital status-wise, single individuals are OR = 0.734 (CI 0.292–1.850 *p* = 0.513) times less likely to have good knowledge compared to married individuals (*p* = 0.05). Similarly, compared to females, males are OR = 0.898 (CI 0.564–1.429 *p* = 0.649) times less likely to have good knowledge about PA, but this difference is not statistically significant (*p* > 0.05). First-year students are OR = 0.928 (CI 0.386–2.236 *p* = 0.868) times less likely to have good knowledge, second-year students are OR = 1.337 (CI 0.565–3.164 *p* = 0.509) times, third-year students are OR = 1.791 (CI 0.784–4.091 *p* = 0.167) times, and fourth-year students are OR = 1.565 (CI 0.763–3.210 *p* = 0.0.222) times more likely to have good knowledge compared to fifth-year students, but this difference is not statistically significant (*p* > 0.05). Compared to individuals with a BMI above 30 (obese), individuals with a BMI <18.5 are OR = 0.77 (CI 0.146–4.061 *p* = 0.758) times more likely to have good knowledge, those with a BMI of 18.5–24.9 are OR = 0.424 (CI 0.107–1.677 *p* = 0.221) times more likely to have good knowledge, and those with a BMI between 25 and 29.9 are OR = 1.476 (CI 0.316–6.889 *p* = 0.621) times more likely, but this difference is not statistically significant (*p* > 0.05). Among the independent variables, the regression results show that none of the variables have a significant effect on good knowledge levels. Details are provided in Table 6.

**Table 6:** Multivariable logistic regression analysis of sociodemographic variables association with knowledge, attitude and practice score.

Variables	Knowledge					Attitude					Practice				
	B value	OR	SE	95% CI	*p*- value	B value	OR	SE	95% CI	*p*- value	B value	OR	SE	95% CI	*p*- value
**Gender**															
Male	−0.108	0.898	0.237	0.564–1.429	**0.649**	−0.750	0.473	0.258	0.285–0.783	0.004	0.665	1.945	0.244	1.207–3.135	0.006
Female	Reference														
**Age**															
18–20 years	−0.066	0.936	0.882	0.166–5.271	0.940	0.211	1.235	1.137	0.133–11.468	0.853	1.988	7.304	1.131	0.796–67.057	0.079
21–25 years	−0.161	0.851	0.869	0.155–4.670	0.853	−0.335	0.716	1.130	0.078–6.550	0.767	1.971	7.181	1.124	0.793–65.009	0.079
Above 25 years	Reference														
**Marital status**															
Single	−0.309	0.734	0.471	0.292–7.850	0.853	−0.956	0.385	0.590	0.121–1.222	0.105	−0.503	0.605	0.578	0.195–1.878	0.385
Married	Reference														
**Year of study**															
First year	−0.074	0.928	0.448	0.386–2.236	0.868	−1.287	0.276	0.518	0.100–0.762	0.013	0.128	1.137	0.450	0.470–2.748	0.776
Second year	0.290	1.337	0.439	0.565–3.164	0.509	−0.654	0.520	0.514	0.190–1.426	0.204	0.141	1.152	0.432	0.494–2.686	0.744
Third year	0.583	1.791	0.421	0.784–4.091	0.167	−1.112	0.329	0.474	0.130–0.832	0.019	0.738	2.091	0.415	0.927–4.715	0.075
Fourth year	0.448	1.565	0.367	0.763–3.210	0.222	−0.742	0.476	0.428	0.206–1.1022	0.083	0.179	1.196	0.366	0.583–2.452	0.625
Fifth year	Reference														
**BMI**															
<18.5	−0.261	0.770	0.848	0.146–4.061	0.758	−0.203	0.816	1.246	0.071–9.381	0.870	1.479	4.388	0.906	0.743–25.901	0.103
18.5–24.9	−0.858	0.424	0.702	0.107–1.677	0.221	−1.344	0.261	1.083	0.031–2.179	0.215	2.313	10.101	0.800	2.104–48.496	0.004
25–29.9	0.389	1.476	0.786	0.316–6.889	0.621	−0.706	0.494	1.136	0.053–4.573	0.534	1.143	3.137	0.842	0.603–16.329	0.174
>30	Reference														

OR, odds ratio.

CI, confidence interval.

BMI, body mass index.

SE, standard error.

B value, Beta value.

##### 3.1.5.1 Attitude toward PA

When compared to the reference age group of over 25 s, the odds of having a positive attitude level increase by OR = 1.235 (CI 0.133–11.468 *p* = 0.853) and decrease by OR = 0.716 (CI 0.078–6.550 *p* = 0.767) times for those between the ages of 18 and 20, and those between 21 and 25, respectively. However, since *p* > 0.05, this difference is not statistically significant. Marital status-wise, single individuals are OR = 0.385 (CI 0.121–1.222 *p* = 0.105) times less likely to have positive attitudes compared to married individuals (*p* > 0.05). Similarly, compared to females, males are OR = 0.473 (CI 0.285–0.783 *p* = 0.004) times less likely to have a positive attitude about PA and this difference is statistically significant (*p* < 0.05). First-year and third-year students are, respectively, OR = 0.329 (CI 0.130–0.832 *p* = 0.013) and OR = 0.276 (CI 0.100–0.762 *p* = 0.019) less likely to have a positive attitude (*p* < 0.05). However, second-year and fourth-year students are, respectively, OR = 0.520 (CI 0.190–1.426 *p* > 0.204) and OR = 0.476 (CI 0.206–1.102 *p* = 0.083) times less likely to have a positive attitude compared to fifth-year students (*p* > 0.05). Compared to individuals with a BMI above 30 (obese), individuals with a BMI <18.5 are 0.816 times (*p* = 0.870) less likely to have a positive attitude, those with a BMI between 18.5 and 24.9 are OR = 0.261 times (CI 0.031–2.179 *p* = 0.215) less likely, and those with a BMI between 25 and 29.9 are OR = 0.494 (CI 0.053–4.573 *p* = 0.534) times less likely, but these differences are not statistically significant (*p* > 0.05). Among the independent variables, the regression results show that being male and being a first-or third-year student has a significant effect on positive attitude levels. Details are provided in Table 6.

##### 3.1.5.2 Practice toward PA

When compared to the reference age group of over 25 s, the odds of having a satisfactory PA level increase by approximately OR = 7.304 and OR = 7.181 (CI 0.796–67.057 and CI 0.793–65.009 *p* = 0.079) times for those between the ages of 18 and 20, and 21 to 25, respectively. However, since *p* > 0.05, this difference is not statistically significant. Marital status-wise, single individuals are OR = 0.605 (CI 0.195–1.878 *p* > 0.385) times less likely to have satisfactory PA practice compared to married individuals (*p* > 0.05). Compared to females, males are OR = 1.945 (CI 1.207–3.135 *p* = 0.006) times more likely to have satisfactory PA practice. *p* < 0.05 indicates that this difference is statistically significant.

First-year students are OR = 1.137 (CI 0.470–2.748 *p* = 0.776) times more likely to have satisfactory practice, second-year students are OR = 1.152 (CI 0.494–2.686 *p* = 0.744) times more likely, third-year students are OR = 2.091 (CI 0.927–4.715 *p* = 0.075) times more likely, and fourth-year students are OR = 1.196 (CI 0.583–2.452 *p* = 0.625) times more likely to have satisfactory practice compared to fifth-year students, but these differences are not statistically significant (*p* > 0.05). Compared to individuals with a BMI above 30 (obese), individuals with a BMI <18.5 are OR = 4.388 (CI 0.743–25.901 *p* = 0.103) times more likely to have satisfactory PA practice, those with a BMI of 18.5–24.9 are OR = 10.101 (CI 2.104–48.49 *p* = 0.004) times more likely to have satisfactory practice, and those with a BMI between 25 and 29.9 are OR = 3.137 (CI 0.603–16.329 *p* = 0.174) times more likely. Among these BMI groups, only the 18.5–24.9 group is found to be significant (*p* < 0.05). Among the independent variables, the regression results show that being male and having a BMI between 18.5 and 24.9 has significant effects on satisfactory PA levels. Details are provided in Table 6.

## 4 Discussion

The purpose of this study was to investigate the current KAP toward PA among healthcare students at King Khalid University of Abha, Saudi Arabia. The majority of participants, 264 (68.9%), showed a good understanding of PA, 288 (75.2%) had a positive attitude toward PA, and 242 (63.2%) had satisfactory PA practices. The study’s findings showed that although these students’ knowledge and attitudes toward physical exercise were generally good, their actual practices were less impressive than their knowledge and attitudes. In earlier research conducted in the U.S., 49.9% of students reported moderate to intense exercise, and 39.1% of respondents engaged in at least moderate activity (24, 25). Hence, compared to students in the United States, our study participants were more likely to engage in high levels of PA. Research by Moini et al. (26) and Sanaee et al. (27) revealed conflicting results with the present study, which could be clarified by the low level of PA knowledge, according to a review of the literature. According to another study, the majority of females had low knowledge (64%) and practice scores (72.2%), but they also had positive attitudes (75.1%) toward physical exercise (19). In this study, 36.8% of the subjects had poor practice results, suggesting that physical inactivity is quite prevalent among the participants. Furthermore, a high degree of physical inactivity was found in previous KSA-conducted research (17, 28). Undoubtedly, the rising prevalence of chronic diseases and physical inactivity can be attributed to the Saudi population’s changing lifestyle (29, 30). Exercise is a powerful tool for maintaining health and preventing illness. Increasing public awareness of the benefits of PA could help avoid obesity and chronic illnesses (31).

In our study, 33.9% of students were physically inactive: that did not do moderate-intensity sport for 30 min, more than five times a week (such as dancing, brisk walking, or light exercise). Only 40.7% stated that they played sports with a high level of intensity for 20 min, three to five times a week, while 38.9% stated that they did not. In another study, 31.8% (32) were found to be physically inactive. This variation may be related to the inclusion of university students, who tend to engage in less exercise due to their academic responsibilities. In a prior study, 31–51% of participants reported doing less than 2.5 h of PA per week (32).

The WHO recommends 60 min or more of moderate to intense physical exercise per day for adolescents, although the majority of teenagers worldwide do not meet this recommendation (33). The majority of students do not engage in daily PA at the suggested levels, according to this study. Only 44% of male adolescents and 20% of female adolescents in Saudi Arabia were found to be sufficiently active in a prior study (28).

A lack of understanding and practice patterns among children, teenagers, and adult Saudi subjects may also be reasons for the comparatively low level of physical exercise (34–36). According to one study, Saudis do not engage in PA for a sufficient duration or at a consistently adequate level. This could be due to a variety of factors, including ignorance, and a lack of time, resources, and motivation (37–40). In contrast to our findings, Ziari et al. (32) found that their participants’ knowledge of PA and attitude toward PA were significantly low. In order to promote PA on campus, it appears that the integration of implementation intentions with e-health platforms and the application of customized and comprehensive intervention models may prove sustainable (41, 42).

It is also important to recognize cultural norms and challenges to exercise, especially for college students, as well as approaches for encouraging exercise training, given the critical role that regular PA plays in human well-being. This is because future medical and paramedical students will be responsible for community care and health promotion. Therefore, more research is needed to ascertain the degree of PA and to provide students with promotional strategies for exercising. It appears that the following strategies can increase the amount of PA that students engage in daily: incorporating regular exercise regimens into curricula as mandatory course units; planning different sports events and classes in residences; providing guidance and education through educational programs; and offering suitable space with areas set aside for PA.

## 5 Limitations

The brief study period and the lack of anthropometric parameters in the questionnaire were two of the study’s limitations. To determine the KAP level, a self-reported questionnaire was also employed. The drawbacks of a cross-sectional survey study design include the lack of data on attitudes over time, the potential for recollection bias, and an inability to demonstrate causation. Because the snowball sampling strategy depends on recommendations to find participants, sample bias may arise.

## 6 Conclusion

The majority of participants in our study showed good knowledge and good attitudes toward PA, but a lower number of participants had satisfactory PA levels. Health education and health promotion initiatives should maintain and enhance knowledge of the positive effects of PA on health. Increased efforts are needed to improve KAP toward PA and to offer chances for regular, structured PA. It is advised to implement coordinated community and educational initiatives. Enhancing PA opportunities for students would require government and school-based policies to establish guidelines. In the future, health education campaigns must create awareness of PA in the student community. Collaboration with institutions and facilities will increase awareness of existing PA programs and help design campaigns. On-campus PA events such as fitness challenges, group exercise sessions, and health fairs can be conducted regularly to encourage participation and raise awareness.

## 7 Future implications

To raise awareness of PA among students, it is imperative that healthcare providers (HCPs) provide advice on future health education programs. HCPs collaborating with institutions and facilities will develop campaigns and raise awareness of the PA initiatives that are currently in place.

## Data Availability

The original contributions presented in the study are included in the article/supplementary material, further inquiries can be directed to the corresponding author.
